# Hydrastis Canadensis in Uterine Troubles

**Published:** 1888-02

**Authors:** Robt. B. Barron

**Affiliations:** Clinton, Georgia


### HYDRASTIS CANADENSIS IN UTERINE TROUBLES.*

BY ROBT. B. BARRON, M. D., CLINTON, GEORGIA.

*Case 1.*—White; twenty-seven years old; has been married twelve years; was never pregnant; has had more or less trouble with her menses ever since she began to menstruate.

This lady has been under my treatment for retroversion and congestive dysmenorrhœa since July, 1886.

I replaced the uterus and kept it in position for sometime with tampons, made of pledgets of cotton soaked in carbolic acid

* Read before the Medical Association of Georgia. From the Transactions.

and glycerine; afterwards fitted a pessary—Thomas' modification of Cutter's—and she has worn it for several months without any discomfort and with a certain amount of relief.

After relieving the displacement, profuse leucorrhœa set in, the menstrual flow coming on every two or three weeks, being very profuse, and lasting for eight or ten days, accompanied with very violent pains in the region of the ovaries, with portions of membrane being thrown off, very closely resembling the decidua.

I had resorted to ergot and black haw, to tonics of all kinds, to the valærianates, in the desperate hope that something might be accomplished, but to find on each visit that my patient was growing worse and weaker, and with the full consciousness that unless something was done she could not long survive.

I made as thorough examination with the speculum of the uterus and its appendages as I could, as I had done before on several occasions, by placing the lady in Sims' position and by by-manual palpation and other means.

I found great pain over the region of the ovaries, the cervix somewhat swollen and congested, the uterus in about the normal position, having worn a pessary for over four months. I introduced the uterine sound and found the depth of the uterine canal to be three and one-half inches. I concluded the trouble must be with the endometrium. I introduced the sponge-tube to dilate the cervical canal until I could thoroughly curette the uterus, removing considerable villous degeneration. I then plugged the uterus with cotton, soaked in the tincture of iodine, and made local applications of the tincture twice a week, as recommended by Mundé in a recent number of the *New York Medical Journal.* Under this treatment she seemed to improve until the next flow, when her condition was worse than ever. I was about disgusted with the case and its treatment, when I saw in the *New York Medical Journal*, for February 19th, 1887, an article on Hydrastis Canadensis in Uterine Hemorrhage, by Dr. Reynold W. Wilcox. On the twenty-third of February I put the lady on the fluid extract of hydrastis, twenty drops an hour after each meal, with another dose at eleven o'clock at night in a wineglass of water. I have seen the patient every week since. Her condition is markedly improved; her menses have made their appearance but twice since. She has suffered but little with the pains in the region of the ovaries. Her menstrual flow is about natural in quantity and lasts for four or five days, and the leucorrhœa has entirely disappeared.

*Case 2.*—A colored woman, thirty-six years old; has been the rounds of the doctors for treatment of uterine troubles; has had three children and miscarried five or six times according to her statement, the last time about two years ago. I saw the patient about six months ago; her general condition was very poor. On examination I found the perineum partially ruptured, together with a lacerated cervix; the womb was anteverted; there was considerable menstrual flow every two weeks, passing lumps of clotted blood; the region of the ovaries and fallopian tubes were very sore and tender. I operated successfully on the perineum, and introduced a Thomas anteversion pessary and got the womb into tolerably good position; still the bleeding continued profuse. After using all the remedies of which I was master, I then treated her by curetting the uterus and by the local application of nitrate of silver 1ʒ to 1℥ of water. This did but little or no good. I have recently let all local treatment alone, only to insist on her wearing the pessary, and have been giving hydrastis, as in the first case, and the patient has steadily improved.

*Cases 3 and 4.*—Whites; aged respectively 45 and 46 years. Both have been troubled with climacteric bleeding for the last year or two, having profuse hemorrhage at times, together with large lumps of clotted blood. I recommended that they keep quiet at the times of their flow and take twenty-drop doses of hydrastis four times a day. They have both been following this treatment for two months; both have greatly improved, and they say they feel better than they have for over a year.

I know my number of cases is small, and will be accepted as proving very little, yet I believe hydrastis to be the best drug known to the profession at present to control uterine hemorrhage due to congestive dysmenorrhœa. Subinvolution from endometritis, metritis and at the climacteric.

